# A model for psychiatric nurses to facilitate the mental health of women living with borderline personality disorder

**DOI:** 10.4102/curationis.v44i1.2157

**Published:** 2021-01-15

**Authors:** Nompumelelo Ntshingila, Annie Temane, Marie Poggenpoel, Chris Myburgh

**Affiliations:** 1 Department of Nursing, Faculty of Health Sciences, University of Johannesburg, Johannesburg, South Africa; 2 Department of Educational Psychology, Faculty of Education, University of Johannesburg, Johannesburg, South Africa

**Keywords:** psychiatric nurses, model, mental health, borderline personality disorder, facilitation of self-empowerment

## Abstract

**Background:**

Borderline personality disorder (BPD) is characterised by emotional dysregulation, feelings of worthlessness, impulsivity, suicidality and poor relationships. As a result of the challenges in the treatment of women living with BPD and the lack of skills from the psychiatric nurse, there was a need to develop a model for psychiatric nurses to facilitate the mental health of women living with BPD.

**Objectives:**

To describe the process that was followed in developing, describing and evaluating a model that could be used as a framework of reference for psychiatric nurses to facilitate the mental health of women living with BPD.

**Method:**

A theory-generative, qualitative, exploratory, descriptive and contextual study design was used to develop the model. The central concept of the model was derived from a previous study: ‘The experiences of women living with borderline personality disorder’. The process entailed the identification of the central concept and other essential criteria, the classification of the central concepts and describing the relationships between the concepts.

**Results:**

The central concept was identified as ‘facilitation of self-empowerment’ of women living with BPD. The concepts ‘facilitation’ and ‘self-empowerment’ were defined and classified. The identified and defined central concepts were placed into interrelated statements. The model to facilitate self-empowerment of women living with BPD was developed, described and evaluated. The model has not been implemented.

**Conclusion:**

The model provides a framework of reference for psychiatric nurses to facilitate self-empowerment of women living with BPD.

## Introduction

Borderline personality disorder (BPD) is a pervasive pattern of instability of interpersonal relationships, self-image, affects and marked impulsivity that begin by early adulthood (American Psychiatric Association [APA] 2013:663). There are nine criteria of BPD according to the Diagnostic and Statistical Manual of Mental Disorders 5 (DSM 5). These criteria are feelings of abandonment, patterns of unstable and intense relationships, identity disturbance, impulsivity, recurrent suicidal behaviour, affective instability, chronic feelings of emptiness, inappropriate intense anger and transient, stress-related paranoid ideation (APA 2013:663). The prevalence of BPD in the United States of America’s general population is 1.6% (Skodol 2017:np). In the Netherlands, 1.1% of the population reported more than five BPD criteria (Ten Have et al. 2016:13). In a retrospective record review conducted at a Johannesburg psychiatric ward in South Africa in 2010, 18.5% of their patients were admitted with BPD.

In the majority of clinical settings, BPD is mostly diagnosed in women with a ratio of 1:4 for men (APA 2013:664). Cahn (2014:1) wrote a paper on women and BPD and suggested that women with BPD are the ‘most difficult patients’, and unresponsive to psychopharmacology or psychotherapy. In the same study, it was demonstrated that many clinicians were struggling with finding empathy for and optimism toward people meeting the diagnostic criteria for BPD. In another study by Bodner et al. (2015:2), it is stated that clinicians experience having limited skills and confidence in the area, thus leading to poor outcomes which, in turn, reinforced their negativity toward women living with BPD. These poor outcomes include the revolving door of hospitalisations, high dropout rates, antagonisation of staff and suicide threats (Bodner et al. 2015:2). These studies indicate how the challenges from clinicians impact on the patients with BPD outcomes.

Psychiatric nurses working with women living with BPD have been said to experience distress, anxiety or confusion, and burnout at some point in their professional lives (Cambanis 2012:102). This is because psychiatric nurses being at the frontline around the clock with these affected patients need more skills in dealing with such patients (Bodner et al. 2015:3). Psychiatric nurses perceive management difficulties associated with patients with BPD and label these patients as ‘attention-seeking’ and ‘acting-out’. The harsh attitudes from psychiatric nurses are deemed unhelpful (McNee, Donoghue & Coppola 2014:35). This can be attributed to the poor attitude towards people with BPD, and the lack of skills and knowledge to manage patients with BPD (Dickens, Hallet & Lamont 2015:3). Sansone and Sansone (2013:41) attribute the poor attitude of psychiatric nurses to a normal human reaction to the complex and pathological behaviours of patients with BPD. The lack of skills and knowledge specifically relates to the management of the challenging dynamics of patients with BPD. In another study conducted by O’Connell and Dowling (2013:27), it was evident that psychiatric nurses require specific skills when working with clients with BPD and the absence of these specific skills was particularly difficult amongst the participants. The skills that psychiatric nurses lacked were the ability to foster and maintain a good therapeutic relationship and to understand and respond to the service users’ emotions and worldview (Roth & Pilling 2013). Because of these challenges, many women living with BPD are unable to access appropriate care, which can worsen their course and prognosis (Links, Ross & Gunderson 2015:753).

These studies highlight the psychiatric nursing challenges in managing patients with BPD. The researchers of this study were then interested in what the women living with BPD were experiencing. A study was conducted focusing on the experiences of women living with BPD (Ntshingila et al. 2016) in Johannesburg, South Africa. This was to ascertain the realities that these women were experiencing and to get a holistic picture as there was a lot of research conducted with clinicians and psychiatric nurses. From this study, one of the themes that came forth was that the women wanted facilitated mental health from the psychiatric nurses because they spend most of the time with psychiatric nurses when they were admitted to the psychotherapy ward.

### Problem statement

Authors found that women living with BPD despite the challenges needed to be assisted with the mental health from the psychiatric nurse (Ntshingila et al. 2016:117). The women living with BPD expressed that through their challenging experiences of living with BPD they yearned for facilitated mental health. Yearning for facilitated mental health meant that the women living with BPD yearned for a change in their ways of living and wanted to see positive progress in their lives (Ntshingila et al. 2016:117). The women living with BPD acknowledged that they would need someone who would walk the journey with them in making this change and it was clear that the psychiatric nurse would be the ideal person to walk this journey with them. The authors were aware of the challenges that were raised from the literature about psychiatric nurses working with patients with BPD. The authors also acknowledged information from the literature that psychiatric nurses expressed interest in learning short-term interventions to overcome the challenges they experience when working with patients diagnosed with BPD (Bodner et al. 2015:9). Despite the challenges that psychiatric nurses experience as well as the challenging experience of women living with BPD, the researchers saw fit that a model for psychiatric nurses to facilitate the mental health of women living with BPD was developed, described and evaluated.

There are existing models developed internationally that were found in the literature on the topic of BPD. Mortimer-Jones et al. (2019:972) developed an Open Borders program. This program provides brief admission, respite and phone-coaching for people with BPD who are primarily reliant on the public mental health system. The purpose of this program was to move away from the medical model, as it is based in a nursing-led residential facility with no medical staff on site. Another model was an educational intervention model to change mental health nurses’ attitudes about people diagnosed with BPD (Dickens et al. 2019). The shortcomings of these two models are that they are not developed to assist psychiatric nurses to facilitate the mental health of women living with BPD.

There is no existing model that can be used as a framework of reference to facilitate the mental health of women living with BPD in South Africa. Stroud and Parsons (2013:250) explored the community psychiatric nurses’ experiences of working with patients diagnosed with BPD. From this study, the findings indicated that when psychiatric nurses have a framework to explain behaviour, they were more likely to express positive attitudes and when they did not have such a framework, they were more likely to view clients in more pejorative terms. To address this gap, a model for psychiatric nurses as a framework of reference to facilitate the mental health of women living with BPD in a public psychiatric hospital was developed, described and evaluated.

### Research purpose

The purpose of this research study is to describe the development of a model as a framework of reference for psychiatric nurses to facilitate the mental health of women living with BPD.

## Definition of central concepts

### Model

A model is a symbolic representation of empiric experience in the form of words, pictorial or graphic diagrams, mathematical notations or physical structure. It is a form of knowledge within the empirical pattern (Chinn & Kramer 2011:157). In this article, a model to serve as a framework of reference for psychiatric nurses to facilitate the mental health of women living with BPD was developed.

### Psychiatric nurse

Psychiatric nurse is a professional nurse registered with the South African Nursing Council as a psychiatric nurse who has received a diploma or a baccalaureate qualification. This nurse works in a specialised setting to provide the bulk of the nursing care to the patients. They have a major responsibility to the public and have contact with patients at all stages of life (Kniesl & Trigoboff 2013:20). The psychiatric nurse directs his or her efforts towards the promotion of mental health and the prevention of mental disturbances (Uys & Middleton 2014:37). In this article, psychiatric nurses worked in the psychotherapy unit. The psychiatric nurse had the responsibility for the promotion, prevention and intervention of mental health of women living with BPD.

### Facilitation

According to the University of Johannesburg (2017:7), facilitation is a dynamic and interactive process for the promotion of health by creating a positive environment and mobilising resources, as well as identifying and bridging obstacles in the promotion of health. In this article, the researchers developed a model as a framework of reference for psychiatric nurses to facilitate the mental health of women living with BPD.

### Mental health

This is a state of well-being in which every individual realises his or her own potential, can cope with the normal stresses of life, can work productively and fruitfully, and is able to make a contribution to her or his community (World Health Organization [WHO] 2014:1). In this article, mental health refers to coping with everyday life by women living with BPD.

### Borderline personality disorder

The DSM 5 (APA 2013:646) states that BPD is a severe psychiatric disorder characterised by a pattern of instability in interpersonal relationships, self-image, affects and marked impulsivity. In this article, the model was developed, described and evaluated for women living with BPD.

## Development of a model to facilitate the mental health of women living with borderline personality disorder

The steps of Chinn and Kramer (2011:163–182) for theory development were used. These steps are concept analysis, relationship statements, description of the model and the evaluation of the model.

### Step 1: Concept analysis

Concept analysis took place in two phases. In phase one, the central concept was identified and in phase two the identified concept was defined and classified.

#### Phase one: Concept identification

In order to identify concepts as building blocks for the model, the researcher used an inductive theory-generative research design (Chinn & Kramer 2011:216). The research design and procedures will be discussed below to describe how the concepts were identified.

**Research design:** In order to develop a model, an inductive approach (Chinn & Kramer 2011:216) was adopted together with a qualitative, exploratory, descriptive and contextual research design (Chinn & Kramer 2011:216; Holloway & Wheeler 2010:2; Ravitch & Carl 2016:68,148).

**Ethical consideration:** The researchers obtained permission to conduct the study from University of Johannesburg Academic Ethics Committee and Higher Degrees Committee, and University of the Witwatersrand Human Research Ethics Committee (medical) – (reference numbers: AEC-01-56-2014, HDC-01-55-2014 and M150249). Permission was also obtained from the Chief Executive Officer of the public psychiatric hospital where the research was conducted. Ethical principles of respect for autonomy, non-maleficence, beneficence and justice (Dhai & McQuoid-Mason 2011:13–14) were taken into consideration. The principle of respect for autonomy was respected through allowing the women living with BPD to make an informed decision before signing an informed consent form; their confidentiality was respected throughout the research by not exposing their names. The participants participated in the study voluntarily and could withdraw their participation at any time during the study. Privacy was ensured to all the participants during the interviews. The principle of non-maleficence was ensured through providing debriefing sessions to participants when required. The researcher being sensitive during the interviews and avoiding harm and discomfort to the participants ensured the principle of beneficence. The researcher ensured the principle of justice through the selection of the participants fairly and without discrimination.

**Population and sample:** The population included all women between the ages of 18–40 years who were diagnosed with BPD. Purposive sampling of eight women diagnosed with BPD was performed, based on the following inclusion criteria: aged between 18 and 40 years, diagnosed with BPD and those who were admitted to the psychotherapy ward in a public psychiatric hospital in Johannesburg. The participants between the ages of 18–40 years were chosen because in a study conducted by Biskin (2015:304), it is reported that the course of BPD during adolescence is not very stable and there is an absence of evidence regarding the course and outcomes of patients who do meet the full criteria for BPD in childhood. The cut-off limit of 40 years old was chosen because in a 16-year prospective follow-up study conducted by Zanarini et al. (2012:480), it is stated that most patients with BPD improve with time and as the participants grew older they no longer met the criteria for BPD.

**Data generation procedure:** The first author conducted in-depth interviews. These interviews were performed mostly at the public psychiatric hospital where the participants were admitted and some at their homes. The autonomy of the women living with BPD was respected by allowing the participants to make an informed decision before signing an informed consent form; their confidentiality was respected throughout the research by not exposing their names and using codes to identify the interviews. The participants were not under any compulsion to participate in the study and could withdraw from the study at any time. This was indicated in the participant’s information letter. Privacy was maintained by conducting in-depth interviews in a private space. The women living with BPD were asked the central question ‘Tell me your life story?’ Each in-depth interview with the women living with BPD was at least 30–45 min long. Data were collected from the eight in-depth interviews, observational notes during the in-depth interviews and the field notes. The first author made field notes after each in-depth interview. The field notes included accurate descriptions of the experiences of the first author whilst conducting the in-depth interviews, non-verbal communication that is observed from the participants and recording of any other activities, and the interpretation of those activities during the in-depth interviews. The in-depth interviews were transcribed, which included the observational and field notes. Data saturation was achieved at the fifth interview and three additional interviews were conducted to confirm the findings.

**Data analysis:** Data analysis was conducted using Tesch’s method (Creswell 2014:186). This method is used to organise the data collected from the in-depth interviews into data that have meaning. Data were coded by developing and applying a list of codes to new segments of data each time an appropriate segment was encountered. The first author and an independent coder had a consensual discussion about the themes and categories that were derived.

**Measures to ensure trustworthiness:** Trustworthiness is the degree of confidence that researchers have in their data. Measures of trustworthiness were assessed by using the criteria of credibility, transferability, dependability and confirmability (De Vos et al. 2011:419–420). All of these criteria were applied to this study.

Credibility was ensured by prolonged engagement with the women living with BPD. The in-depth interviews were approximately 45–60 min. Triangulation of data was performed using multiple data collection methods. An independent coder, who was knowledgeable about the analysis of qualitative data was consulted, and consensus was reached on the identified themes and categories in the data. The research results and model structure was presented at research forums and doctoral seminars, and corrections and modifications were made based on the recommendations of experts in research and model development.

Transferability was ensured through extensive description of the demographics of the women living with BPD and similarly a description of findings supported by direct quotations (Ntshingila et al. 2016).

Dependability was ensured through a detailed description of the research methodology. A dependability audit was conducted in which the three supervisors evaluated the study before it was presented to external assessors to ensure dependability.

The researchers monitored the whole research process for accuracy and relevancy of data. The researchers kept an accurate record of the research process of the study so that other researchers could emulate the process and reach a similar conclusion. An independent coder with expertise in qualitative research was used to collate the findings.

**Findings of the study:** The findings of this study indicated that there were childhood experiences such as living in an unsafe space as a result unhealthy family dynamics, boundary violations and educational challenges. These women living with BPD experienced chronic feelings of emptiness in relationships with the self. They also presented with a pattern of unstable interpersonal relationships and compromised mental health, which was apparent through the early onset of mental problems, emotional upheavals, longings for emotional escape and having different trigger factors. These women yearned for facilitated mental health (Ntshingila et al. 2016:110–119)

**FIGURE 1:**
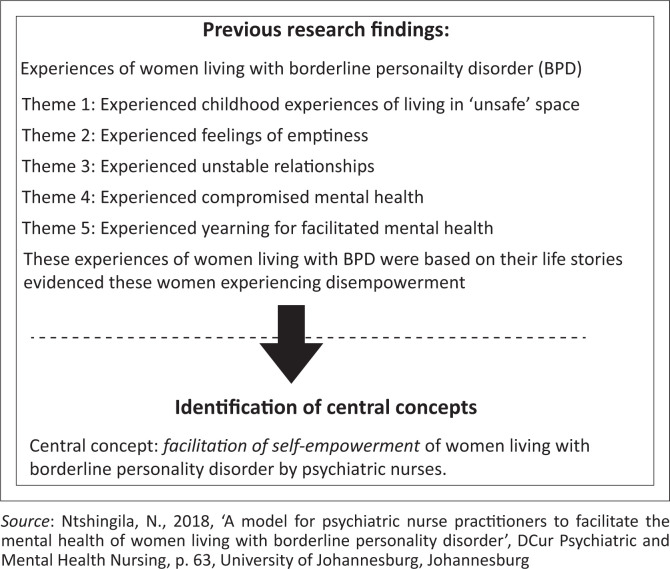
Identification of the central concept.

**Identification of the central concept:** The central concepts were derived from the findings of the researchers’ previous study (Ntshingila et al. 2016:110–119) (see Figure 1 for identification of the central concepts from the findings of the experiences of women living with BPD). The themes were used in identifying the central concept. The experiences of women living with BPD indicated that these women were disempowered. *Facilitation of self-empowerment* as a central concept was identified.

#### Phase two: Definition and classification of the central concept

During this phase, the concept was given meaning (Chinn & Kramer 2011:177). The definition of concepts was performed connotatively as well as denotatively using dictionaries and available literature (Walker & Avant 2011:161). Essential attributes for the definition of the central concept were identified from dictionary and subject definitions. ‘Facilitation of self-empowerment’ was defined based on the essential attributes as follows (see Table 1) (Ntshingila et al. 2016:437–443).

**TABLE 1:** The essential attributes of the concept ‘facilitation of self-empowerment’.

Central concept	Essential attributes
Facilitation	Assisting the progress of a person
	Dynamic, interactive process
	Creating a positive environment
	Mobilising resources
Self-empowerment	Taking charge of one’s life
	Taking an active role in the journey of self-discovery
	Feeling secure and connected
	Develop a sense of meaning and coherence
	Knowing what is best for you

Source: Ntshingila, N., 2018, ‘A model for psychiatric nurse practitioners to facilitate the mental health of women living with borderline personality disorder’, DCur Psychiatric and Mental Health Nursing, pp. 73–74, University of Johannesburg, Johannesburg

A final definition was formulated, synthesising all the attributes of the definition contained in the dictionary and subject definitions as indicated in Table 1. The definition includes the two central concepts, indicating the relationships amongst the central concepts. In the facilitation of self-empowerment of the women living with BPD, the psychiatric nurse assists in the progress through a dynamic, interactive process by creating a positive environment through mobilising resources in order to promote the mental health of these women. Self-empowerment is achieved when the woman living with BPD knows what is best for herself and takes charge of her own life by taking an active role in the journey of self-discovery and feeling secure and connected by developing a sense of meaning and coherence (Ntshingila et al. 2016:442).

The central concepts were then classified by using Dickoff, James and Wiedenbach’s (1968:425) survey list, which asks: ‘Who is the agent?’, ‘Who is the recipient?’, ‘What is the procedure?’, ‘What is the dynamics?’, ‘What is the context?’ and ‘What is the outcome?’.

The central concept was classified by using Dickoff et al.’s (1968:425) survey list as follows:

- The agent is the person who will make certain that the nursing goal is achieved (Dickoff et al. 1968:425). In this study, the psychiatric nurse worked with the women living with BPD. This is a psychiatric nurse who is committed to facilitating self-empowerment as an integral part of mental health through the assessment, diagnosis, and treatment of mental health problems and psychiatric disorders (Kniesl & Trigoboff 2013:18). The psychiatric nurse is responsible for the facilitation of self-empowerment as an integral part of the mental health of women living with BPD (Ntshingila 2018:76).
- Recipients are the people who interact with the agent (Dickoff et al. 1968:427). It is important to note how the recipients view the activity. The recipients in this study were the women living with BPD. They benefited from the facilitation, which is self-empowerment, as an integral part of mental health (Ntshingila 2018:78).
- The context may include home, work, hospital, community and church, etc. The context of this study was a psychotherapy unit where the women living with BPD were currently admitted (Ntshingila 2018:79).
- The dynamics may be barriers or enhancers of the implementation of the model which were the five themes identified from the experiences of women living with BPD. Women living with BPD experienced living in unsafe spaces as a child, chronic feelings of emptiness in relationship with the self, unstable interpersonal relationships and compromised mental health. The psychiatric nurse was motivated to facilitate the self-empowerment of women living with BPD (Ntshingila 2018:79).
- The procedure was psychiatric nurse using the model as a framework of reference to facilitate self-empowerment as an integral part of mental health. The facilitation of self-empowerment took place in three phases: the relationship phase, the working phase and the termination phase (Ntshingila 2018:79).
- The terminus, which is the outcome, was self-empowered women living with BPD as an integral part of mental health (Ntshingila 2018:80).

### Step 2: Describing relationships between the concepts

The identified and defined central concepts were placed into relationship statements (Chinn & Kramer 2011:180). The identified essential attributes were then used to construct relationship statements. Relationship statements help to give the concepts more clarity and add direction to the understanding of the phenomenon (Towell, Nel & Muller 2015:4). In this study, the relationship statements were formulated as follows.

The psychiatric nurse forms a trusting and therapeutic relationship with the woman living with BPD in a psychotherapy unit by creating a positive environment. The psychiatric nurse will mobilise resources to assist the woman living with BPD to be self-empowered. The psychiatric nurse will assist the woman living with BPD to take charge of her own life in order for the woman to be able to make choices about her life. The woman living with BPD needs to take an active role in the journey of self-discovery to achieve self-empowerment. She needs to feel secure and connected with herself, with others and with her environment to be self-empowered. The woman living with BPD needs to develop a sense of meaning and coherence; to know what is best for herself in order to make decisions that are best for herself. By achieving this, the woman living with BPD will be self-empowered.

### Step 3: Description of the model

The criteria for descriptive components of a model were used in describing the model (Chinn & Kramer 2011:186–196). The criteria used for describing the model as a framework of reference for psychiatric nurses to facilitate self-empowerment of women living with BPD included assumptions, purpose, concepts, definitions and relationships. The structure and processes of the model were also described.

#### Structure of the model

The structure of the model is discussed using the headings as described by Chinn and Kramer (2011:185): purpose of the model, assumptions of the model and process description of the model.

#### Purpose of the model

The purpose of this model is to serve as a framework of reference for psychiatric nurses to facilitate self-empowerment as an integral part of the mental health of women living with BPD.

#### Assumptions of the model

The assumptions of this model are based on the Theory for Health Promotion in Nursing (University of Johannesburg 2017:2–16). The assumptions that underlie this model are reflected in the following statements (University of Johannesburg 2017):

A woman living with BPD is viewed holistically and in interaction with her environment. The environment consists of the internal and external environment. The internal environment comprises of body, mind and spirit dimensions, and the external environment consists of the physical, social and spiritual dimensions. (p. 4)

The psychiatric nurse facilitates the self-empowerment of the woman living with BPD through a dynamic and interactive process within a specific context, that is, the psychotherapy unit.

The psychiatric nurse establishes a therapeutic relationship with the woman living with BPD. The therapeutic relationship is a mutually defined, collaborative and goal-orientated professional relationship (Kniesl & Trigoboff 2013:46). Facilitation of self-empowerment is implemented by creating a positive therapeutic environment for the woman living with BPD, and by mobilising resources to facilitate self-empowerment as integral part of mental health.

The facilitation of self-empowerment of the woman living with BPD happens in the context of the psychotherapy unit.

#### Process description of the model

**FIGURE 2:**
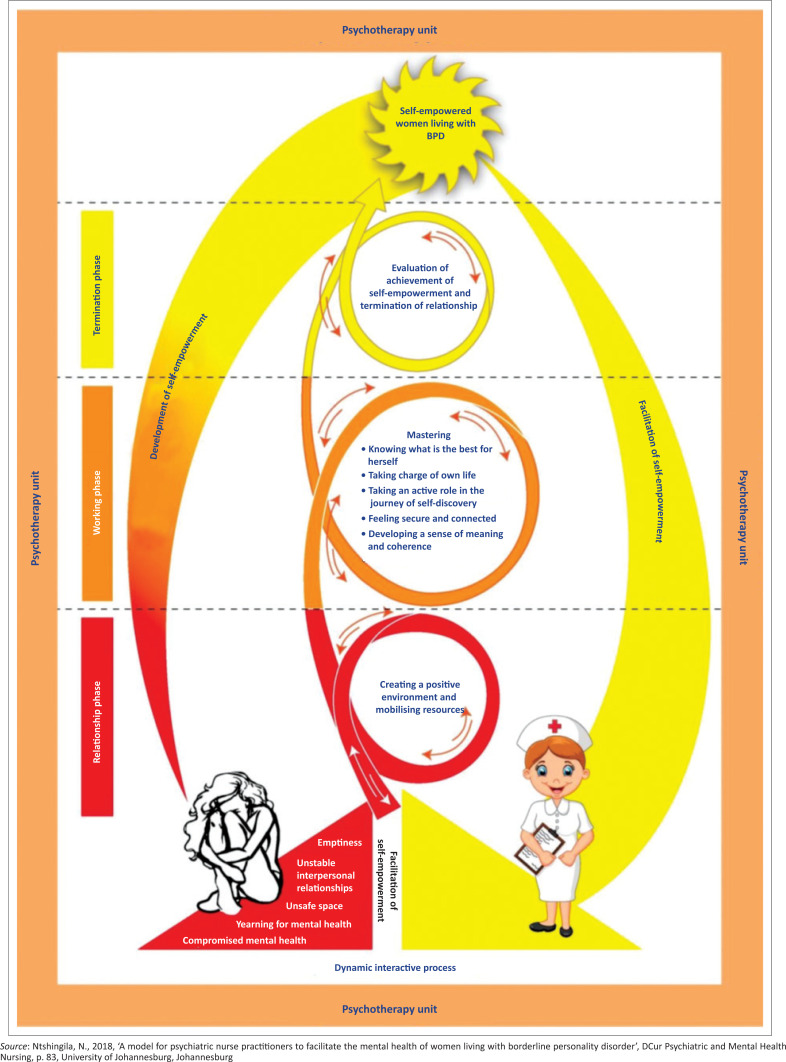
A model to facilitate self-empowerment of women living with borderline personality disorder.

A model for psychiatric nurses to facilitate self-empowerment of women living with BPD is displayed in Figure 2. The context is represented by the peach-coloured border, which indicates the safety of the psychotherapy unit (Empower-yourself-with-colour-psychology 2015). The peach colour is calming and makes one feel safe and secure, unlike the environments that the woman living with BPD has been exposed to in her life.

The woman living with BPD is represented in a red colour on the left-hand side of the model. The colour red in the triangle signifies higher energy levels and increased action (Empower-yourself-with-colour-psychology 2015) which, in the context of this study, is represented by the increase of acting out behaviour of the woman living with BPD, such as suicidal behaviour, mood instability and chaotic interpersonal relationships. The red colour is also applied to the relationship phase of the model because of the emotional status that the woman living with BPD comes in during the relationship phase.

The psychiatric nurse is represented by a triangle in yellow. The colour yellow provides clarity of thought and inspires the thought process (Empower-yourself-with-color-psychology 2015). The psychiatric nurse represents provision of a healing presence of unconditional acceptance, patience, loving kindness, a non-judgemental attitude, understanding, good listening skills, honesty and empathy (Kniesl & Trigoboff 2013:50). All of these qualities are ones the psychiatric nurse should display towards the woman living with BPD. The psychiatric nurse will walk the road with the woman living with BPD.

The facilitation of self-empowerment is indicated with a spiral-shaped diagram. The spiral was chosen as it demonstrates continuity in the process of facilitation by self-empowerment. The spirals also have arrows that are going up and down. The upward-facing arrows indicate the progression through the phases that the woman living with BPD will go through. The upward-facing arrows also indicate the self-empowerment skills that the woman is achieving as she is able to use the coping mechanisms to deal with the experiences of living in an unsafe space, chronic feelings emptiness, unstable interpersonal relationships and compromised mental health. These upward-facing arrows are large in size, indicating that the woman living with BPD achieves self-empowerment. The downward-facing arrows are small in size suggesting the possibility of relapse that the woman living with BPD may encounter. The spiral diagram is red during the relationship phase, orange in the working phase and yellow in the termination phase. These colours denote the phases that the psychiatric nurse is assisting in facilitating the woman living with BPD. The spiral ends with an arrow facing towards the outcome, which is the self-empowered woman living with BPD.

The model consists of three phases, which are the relationship phase, the working phase and the termination phase.

In the relationship phase, the psychiatric nurse establishes a relationship in order to create a positive environment for the woman living with BPD to mobilise resources. The psychiatric nurse will need the skills of creating rapport, safety and trust in order to create a positive environment in the psychotherapy unit (Townsend 2015:129). When the psychiatric nurse creates a positive environment, the outcomes of the mental health patient are improved (Twigg & McCullough 2014:91). The psychiatric nurses mobilise resources through their skills to provide individual therapy, group therapy, mental health education and life-skills training as resources to women living with BPD. The psychiatric nurse is the appropriate person to mobilise these resources because they spend the most time with the woman living with BPD. Once this is achieved, the woman living with BPD should experience that she is in a safe space and can proceed to the working phase.

The next phase is the working phase. In this phase, the psychiatric nurse works with the woman living with BPD to master knowing what is best for her; taking charge of her own life; taking an active role in the journey of self-discovery, feeling secure and connected, and developing a sense of meaning and coherence. The interventions are focused on the woman living with BPD being able to cope with life’s challenges in a thorough manner. The psychiatric nurse assists the woman living with BPD to master knowing what is best for her through gaining awareness of herself and noticing the obvious signs of distress. The woman living with BPD is assisted with awareness exercises, which will enable her to be grounded and to connect with herself. The psychiatric nurse assists the woman living with BPD to take charge of her own life through learning to take responsibility, and learning to identify and acknowledge feelings at a given time. This will assist the woman living with BPD to be in control of how she reacts when uncomfortable feelings are experienced. This control of feelings allows one to feel in charge of one’s life (Klein 2015:2). The psychiatric nurse assists the woman living with BPD to take an active role in the journey of self-discovery. This is performed through a process where the psychiatric nurse assists the woman living with BPD to understand her strengths and weaknesses, what are her goals and how does she plan on achieving those goals (Dunstan 2015:2). The woman living with BPD is assisted in acknowledging fears that are related to achieving her goals and to take an active role instead of a passive role to improve the current situation. The woman living with BPD is empowered to choose the option that will bring about more pleasant feelings (Dunstan 2015:5).

In feeling secure and connected, the psychiatric nurse assists the woman living with BPD to understand that she must reach out when she needs emotional support, understand what her insecurities are and what makes her uncomfortable about those insecurities, and identify professionals whom she trusts and can go to for professional advice. The psychiatric nurse assists the woman living with BPD to develop a sense of meaning and coherence through engaging in activities that are important to her, having an attitude that will impact how well she copes with circumstances, and discussing and developing the coping strategies that seem most appropriate for dealing with stressful situations (Ntshingila 2018).

The last phase is the termination phase. In this phase, there is an evaluation of the achievement of self-empowerment, and the psychiatric nurse evaluates whether the woman living with BPD has achieved self-empowerment. In this phase, the psychiatric nurse also works on terminating the therapeutic relationship that has been built with the affected woman. The woman living with BPD also works on how to take on the mastered skills learned in the process of facilitating self-empowerment to ensure continuity and decreasing chances of relapse. The woman living with BPD needs to ensure that internal and external resources are in place. There is decreased involvement of the psychiatric nurse at this stage to ensure that the woman living with BPD is able to function by herself. This is the indication that the woman living with BPD has achieved self-empowerment.

### Step 4: Evaluation of the model

The model was evaluated using the criteria from Chinn and Kramer (2011:137). These criteria for evaluating a model are clarity, simplicity, generalisability, accessibility and importance (Chinn & Kramer 2011:137). The model was evaluated by a panel of experts in research theory and advanced psychiatric nurses. Experts who evaluated the demographics of the model comprised of four full-time professors (P), one associate professor (AP), three senior lecturers with doctoral degrees (SL) and two lecturers currently busy with their doctoral studies (L). The model was found to comply with the criteria for model development. These criteria are discussed next.

**Clarity:** The clarity of the model is described in terms of semantic clarity, semantic consistency, structural clarity and structural consistency (Chinn & Kramer 2011:198). The panel of experts who evaluated this model stated that the model was structurally clear and the concepts were consistent. The following direct quotations supported the clarity of the model:

‘It explains the aims of facilitating care by building up relationship with client first then working through the identified problem until achievement or termination phase clearly.’ (Participant SL1)

‘The model is clear as it shows the stages that the patient will go through to get self-empowerment.’ (Participant L2)

‘The model is clear as there is alignment to the other steps that is concept analysis.’ (Participant L1)

**Simplicity:** Simplicity describes that the concepts and statements reflected should preferably be minimal (Chinn & Kramer 2011:201). The panel suggested that the model was simple to understand with the explanations given by the researcher. Simplicity of the model was supported by the following direct comments:

‘It describes the main process in the facilitation without complicated concepts.’ (Participant P1)

‘The model is simple and can be easily utilised by psychiatric nurse practitioner and other practitioners in nursing.’ (Participant P4)

‘Very simple and easy to understand.’ (Participant AP1)

**Generality:** Generality is about the breadth and scope of practice of the model (Chinn & Kramer 2011:202). The panel’s suggestions indicated that the model was general enough for the purposes of the model. One suggestion made was that it could be used for any other person who needed self-empowerment, other than women living with BPD. The generality of the model was supported by the following observations:

‘It is suitable to reach objectives and easy to explain.’ (Participant P3)

‘This model can be used for not only for women with BPD by anyone who needs self-empowerment.’ (Participant L1)

‘The model is general and can be easily utilised.’ (Participant AP1)

**Accessibility:** Accessibility addresses the extent to which the empiric indicators can be identified, as well the extent to which the purpose of the model is attained (Chinn & Kramer 2011:203). The model was accessible according to the panel of experts as it was generated from a known theory, which is the Theory for Health Promotion in Nursing (University of Johannesburg 2017). It was also going to be made accessible to the psychiatric nurses. Accessibility of the model was supported by the following observations:

‘It is accessible with regards to the trained professional nurse with appropriate knowledge and adequate workforce and is inexpensive. …’ (Participant P3)

‘It is accessible and could yield positive results.’ (Participant P2)

‘It looks accessible to the professional psychiatric nurse.’ (Participant SL3)

**Importance:** The importance of the model describes the clinical significance and practical value of the model with regard to psychiatric nursing practice, research and education (Chinn & Kramer 2011:204). All the panel members agreed that the model was important in facilitating self-empowerment of women living with BPD. Importance of the model was supported by the following observations:

‘The model is very important for facilitating the mental health of women living with borderline personality disorder.’ (Participant P2)

‘Facilitating mental health is important and the nurses need a guideline for assisting patients to achieve self-empowerment.’ (Participant AP1)

‘It is important so much that nurses working with psychiatric patients will gain knowledge.’ (Participant SL3)

## Limitations

This study has not discussed the implementation of the model and the evaluation of the implementation of the model.

## Recommendations

Recommendations for nursing practice are that the model could be used as a framework of reference in different contexts where there are women living with BPD. The model could be used to promote positive relationships between the psychiatric nurses and the women living with BPD. The model could also be used to enhance the benefits to the psychiatric nurses working with women living with BPD, thus shifting their negative attitudes about women living with BPD. Recommendations for nursing research is that the model could be used for further research in contexts where psychiatric nurses’ experiences could be explored in using this model. In nursing education, the model can be of benefit because it can be included in the undergraduate and postgraduate training of psychiatric nursing students. This could be beneficial to assist psychiatric nursing students to be better equipped to work with women living with BPD.

## Conclusion

Developing a model as a framework of reference for psychiatric nurses to facilitate self-empowerment as an integral part of mental health of women living with BPD provided an original contribution to the theory in Psychiatric and Mental Health Nursing. This model can be used by psychiatric nurses as a tool to facilitate self-empowerment in women living with BPD. The purpose of the article was to describe the development of a model as a framework of reference for psychiatric nurses to facilitate the self-empowerment of women living with BPD. The steps of developing the model were described, along with the ethical considerations and measures to ensure trustworthiness. Limitations and recommendations were also discussed. The original contribution of this study was also discussed.

## References

[CIT0001] American Psychiatric Association (APA), 2013, *Diagnostic and statistical manual of mental disorders*, 5th edn, American Psychiatric Association, Arlington, VA.

[CIT0002] Biskin, R.S, 2015, ‘The lifetime course of borderline personality disorder’, *The Canadian Journal of Psychiatry* 60(7), 303–308. 10.1177/070674371506000702 26175388 PMC4500179

[CIT0003] Bodner, E., Cohen-Fridel, S., Mashiah, M., Segal, M., Grinshpoon, A., Fischel, T. et al., 2015, ‘The attitudes of psychiatric hospital staff toward hospitalization and treatment of patients with borderline personality disorder’, *BMC Psychiatry* 15, a2 10.1186/s12888-014-0380-y PMC4307152 25609479

[CIT0004] Cahn, S.K, 2014, ‘Borderlines of power: Women and borderline personality disorder’, *The Semiannual Newsletter of the Robert Penn Warren Center for the Humanities* 22(2), 1–4.

[CIT0005] Cambanis, E.V.A, 2012, ‘Treating borderline personality disorder as a trainee psychologist: Issues of resistance, inexperience and countertransference, *Journal of Child & Adolescent Mental Health* 24(1), 99–109. 10.2989/17280583.2011.639075 25865841

[CIT0006] Chinn, P.L. & Kramer, M.K, 2018, *Knowledge development in nursing: Theory and process*, 10th edn, Elsevier, St. Louis, MO.

[CIT0007] Creswell, J.W, 2014, *Research design: Qualitative, quantitative & mixed methods approaches*, 4th edn, Sage, London.

[CIT0008] De Vos, A.S., Strydom, H., Fouche, C.B. & Delport, C.S.L, 2011, *Research at grass roots for the social sciences and human service professions*, 4th edn, Van Schaik, Pretoria.

[CIT0009] Dhai, A. & McQuoid-Mason, D, 2011, *Bioethics, human rights and health law: principles and practice*, Juta, Cape Town.

[CIT0010] Dickens, G.L., Hallett, N. & Lamont, E, 2016, ‘Interventions to improve mental health nurses’ skills, attitudes, and knowledge related to people with a diagnosis of borderline personality disorder: Systematic review, *International Journal of Nursing Studies* 56, 114–127. 10.1016/j.ijnurstu.2015.10.019 26747180

[CIT0011] Dickens, G.L., Lamont, E., Mullen, J., MacArthur, N. & Stirling, F.J, 2019, ‘Mixed-methods evaluation of an educational intervention to change mental health nurses’ attitudes to people diagnosed with borderline personality disorder’, *Journal of Clinical Nursing* 28, 2613–2623. 10.1111/jocn.14847 30830704

[CIT0012] Dickoff, J., James, P. & Wiedenbach, E, 1968, ‘Theory in a practice discipline part 1: Practice orientated theory’, *Nursing Research* 17(5), 415–435. 10.1097/00006199-196809000-00006 5186886

[CIT0013] Dunstan, C, 2015, *On a self-discovery and taking an active role in your life*, viewed 07 December 2015, from http://fitting-it-all-in.com/self-discovery-taking-active-role-life

[CIT0014] Empower-yourself-with-color-psychology, 2015, *Meaning of colours*, viewed 15 October 2015, from https://empoweryourself-with-color-psychology.com/meaning-of-colour.html

[CIT0015] Holloway, I. & Wheeler, S, 2010, *Qualitative research in nursing and healthcare*, 3rd edn, Wiley-Blackwell, London.

[CIT0016] Klein, J, 2015, *How to take charge of your life and stop getting in your own way*, viewed 08 December 2015, from https://inspiyr.com/your-life/

[CIT0017] Kniesl, C.R. & Trigoboff, E, 2013, *Contemporary psychiatric-mental health nursing*, 3rd edn, Pearson Education Inc, New Jersey, NJ.

[CIT0018] Links, P.S., Ross, R. & Gunderson, J.G, 2015, ‘Promoting good psychiatric management for patients with borderline personality disorder’, *Journal of Clinical Psychology* 71(8), 753–763. 10.1002/jclp.22203 26197971

[CIT0019] McNee, L., Donoghue, C. & Coppola, A, 2014, ‘A team approach to borderline personality disorder, *Mental Health Practice* 17(10), 33–35. 10.7748/mhp.17.10.33.e887

[CIT0020] Mortimer-Jones, S., Morrison, P., Munib, A., Paolucci, F., Neale, S., Hellewell, A, 2019, ‘Staff and client perspectives of the open borders programme for people with borderline personality disorder’, *International Journal of Mental Health Nursing* 28(4), 971–979. 10.1111/inm.12602 31081282

[CIT0021] Ntshingila, N, 2018, ‘A model for psychiatric nurse practitioners to facilitate the mental health of women living with borderline personality disorder’, DCur Psychiatric and Mental Health Nursing, University of Johannesburg, Johannesburg.

[CIT0022] Ntshingila, N., Temane, A., Poggenpoel, M. & Myburgh, C, 2016, ‘Facilitation of self-empowerment of women living with borderline personality disorder: A concept analysis’, *Health S.A Gesondheid Special Edition* 21, 437–443. 10.1016/j.hsag.2016.09.002

[CIT0023] O’Connell B. & Dowling M, 2013, ‘Community psychiatric nurses’ experiences of working with clients with borderline personality disorder’, *Mental Health Practice* 17(4), 27–33. 10.7748/mhp2013.12.17.4.27.e845

[CIT0024] Ravitch, S.M. & Carl, N.M, 2016, *Qualitative research bridging the conceptual, theoretical and methodological*, Sage, Los Angeles, CA.

[CIT0025] Roth, A.D. & Pilling, S, 2013, ‘A competence framework for psychological interventions with people with personality disorder’, UCL, viewed 14 September 2020, from www.ucl.ac/CORE/.

[CIT0026] Sansone, R.A. & Sansone, L.A, 2013, ‘Responses of mental health clinicians to patients with borderline personality disorder’, *Innovations in Clinical Neuroscience* 10(5–6), 39–43. 23882440 PMC3719460

[CIT0027] Skodol, M.D, 2017, *Borderline personality disorder: Epidemiology, clinical features, course, assessment and diagnosis*, viewed 28 November 2018, from https://www.uptodate.com/contents/borderline-personality-disorder-epidemiology-clinical-features-course-assessment-and-diagnosis

[CIT0028] Stroud, J. & Parsons, R, 2013, ‘Working with borderline personality disorder: A small-scale qualitative investigation into community psychiatric nurses’ constructs of borderline personality disorder’, *Personality and Mental Health* 7(3), 242–253. 10.1002/pmh.1214 24343967

[CIT0029] Ten Have, M., Verheul, R., Kaasenbrood, A., van Dorsselaer, S., Tuithof, M., Kleinjan, M. et al., 2016, ‘Prevelance rates of borderline personality disorder symptoms: A study based on the Netherlands mental health survey and incidence study-2’, *BMC Psychiatry* 16, 249–267. 10.1186/s12888-016-0939-x 27435813 PMC4949762

[CIT0030] Towell, A., Nel, W.E. & Muller, A, 2015, ‘Model of facilitation of emotional intelligence to promote wholeness of neophyte critical care nurses in South Africa’, *Health SA Gesondheid* 20(1), 1–10. 10.1016/j.hsag.2015.04.001

[CIT0031] Townsend, M.C, 2015, *Psychiatric mental health nursing concepts of care in evidence-based practice*, 8th edn, F.A. Davis Company, Philadelphia, PA.

[CIT0032] Twigg, D. & McCullough, K, 2014, ‘Nurse retention: A review of strategies to create and enhance positive practice environments in clinical settings’, *International Journal of Nursing Studies* 51(1), 85–92. 10.1016/j.ijnurstu.2013.05.015 23809644

[CIT0033] University of Johannesburg, 2017, *Department of nursing science paradigm: Theory for health promotion in nursing*, Department of Health Sciences, Johannesburg.

[CIT0034] Uys, L. & Middleton, L, 2014, *Mental health nursing: A South African perspective*, 6th edn, Juta, Cape Town.

[CIT0035] Walker, L.O. & Avant, K.C, 2011, *Strategies for theory construction in nursing*, 5th edn, Prenctice Hall, Upper Saddle River, NJ.

[CIT0036] WHO, 2014, *Mental health*, viewed 24 January 2019, from https://www.who.int/features/factfiles/mental_health/en/.

[CIT0037] Zanarini, M.C., Frankenburg, F.R., Reich, D.B. & Fitzmaurice, G, 2012, ‘Attainment and stability of sustained symptomatic remission and recovery amongst patients with borderline personality disorder and axis II comparison subjects: A 16-year prospective follow-up study’, *The American Journal of Psychiatry* 169(5), 476–483. 10.1176/appi.ajp.2011.11101550 22737693 PMC3509999

